# A case with transient refractive change after removal of pituitary tumor

**DOI:** 10.1186/1471-2415-13-65

**Published:** 2013-11-01

**Authors:** Hiroto Ishikawa, Junsuke Akura, Kazutaka Uchida, Naohiro Ikeda, Tomohiro Ikeda, Cesar V Borlongan, Osamu Mimura

**Affiliations:** 1 Department of Ophthalmology, Hyogo College of Medicine, 1-1 Mukogawa-cyo, Nishinomiya, Hyogo 663-8501, Japan; 2 Department of Ophthalmology, Kushimoto Rehabilitation Center, Kushimoto, Japan; 3 Department of Neurosurgery, Hyogo College of Medicine, 1-1 Mukogawa-cyo, Nishinomiya, Hyogo 663-8501, Japan; 4 Department of Neurosurgery and Brain Repair, University of South Florida, 12901 Bruce B. Downs Blvd, MDC78, Tampa FL 33612, USA

**Keywords:** Refractive change, Trans-sphenoidal surgery, Hyponatremia

## Abstract

**Background:**

Refractive change can be caused by systemic illnesses such as Lupus erythematosus, thyroid deficiency, and diabetes mellitus. However, refractive change after pituitary tumor removal has so far not been reported.

**Case presentation:**

A 62-year-old woman presented with blurred near vision 10 days after trans-sphenoidal surgery (TSS) for a pituitary tumor. Around the same time, she experienced intercurrent hyponatremia. The corrected visual acuity of both eyes was 20/20, the spherical equivalent of the right eye was −2.125 diopters, and of the left eye was −2.0 diopters before TSS. However, 11 days after TSS, the spherical equivalent of the right eye changed to −0.75 diopters, and that of left eye changed to −1.125 diopters without hyperglycemia. There were no changes in the corrected visual acuity during the follow-up.

**Conclusion:**

We demonstrated a case with transient refractive change after TSS. The following mechanism is proposed: Hyponatremia induced by the pituitary tumor removal causes an osmotic change in the aqueous humor with lens swelling. This case report is a reminder to both ophthalmologists and neurosurgeons that ophthalmological factors such as lens thickness and axial length should be taken into account when conducting preoperative examinations especially for patients undergoing TSS.

## Background

Refractive change of the eye is affected by a combination of axial length, corneal power anterior chamber depth and lens power. In general, refraction changes with age. In a recent report about lens power and refractive error, the axial length and the lens power was found to influence the refractive error mostly in adults aged over 50 [1,2].

Refractive change is often described in systemic illness such as Lupus erythematosus [3], thyroid deficiency [4], pregnancy, orbital tumor [5], and diabetes mellitus (DM). Refractive change has been recognized in DM since the 19th century and was well-described throughout the 20th century [6-10].

The pathologic occurrence of refractive change falls under two patterns: one is lens thickness changing due to an abnormal metabolic process, and the second is a change of eye ball shape caused by an increase of orbital contents. Lupus erythematosus and DM cause abnormal water metabolism and influx of aqueous humor from the anterior chamber into the lens, culminating in an increased lens thickness [3,6-10]. Thyroid deficiencies and orbital tumors increase orbital contents due to inflammation or a tumor, compressing the eye ball. As a result, the eye ball shape is altered, which in turn produces refractive change [4,5].

To date, refractive change in hyponatremia after trans-sphenoidal surgery (TSS) has never been reported. In this case, we report the refractive change following TSS, and discuss the causal correlation between refractive change and hyponatremia.

## Case presentation

**Figure 1:**
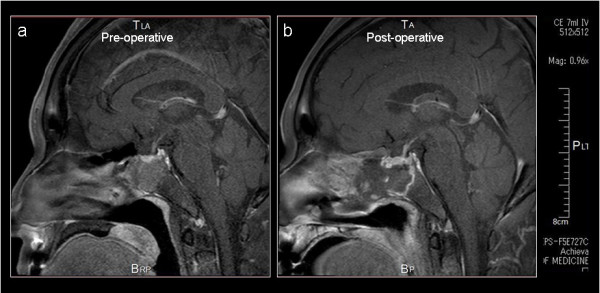
**Brain MRI.** MRI of pre- **(a)** and post-operative pituitary tumor **(b)**.

A 62-year-old woman complaining of dizziness was admitted to our college hospital. The patient had a past medical history of thyroid dysfunction. She was neurologically intact, and had no abnormalities in her blood tests. Magnetic resonance imaging (MRI) showed an extended intrasellar tumor, which measured 25-mm in its greatest diameter and did not attach to the optic chiasm. The lesion infiltrated the right cavernous sinus, and extended to the pharynx (Figure 1a). Initially, the tumor was considered to be a nonfunctional pituitary adenoma.

**Figure 2:**
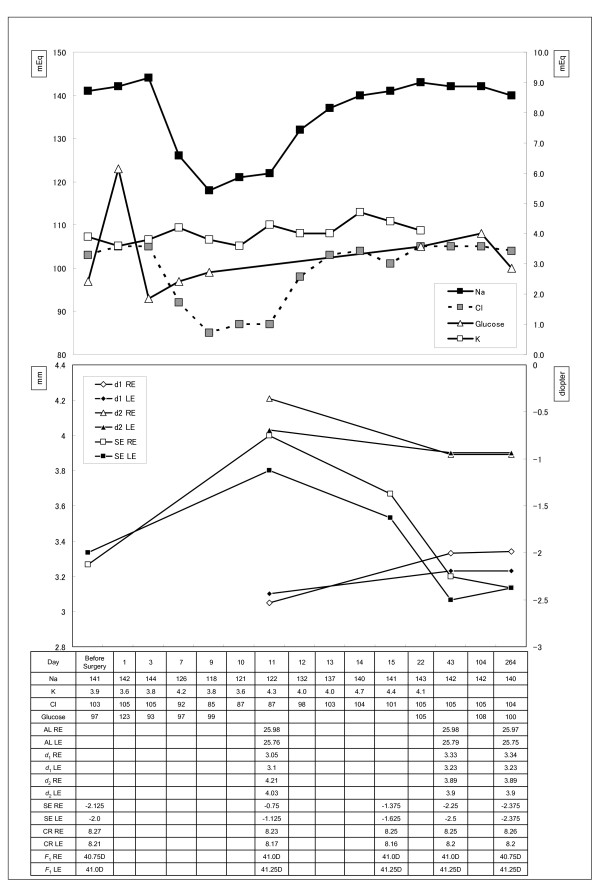
**Patient findings.** The time course of serum electrolyte levels, anterior chamber depth (*d*_1_), lens thickness (*d*_2_), spherical equivalent (SE), axial length (AL), averaged corneal radius (CR) and corneal power (*F*_1_) of both eyes (RE; right eye, LE; left eye). Both spherical equivalent and lens thickness values at onset are higher than at follow up, while in contrast the anterior chamber depth value is lower.

The patient consented to TSS, and TSS was performed without incident (Figure 1b). Seven days after TSS, the patient presented with associated hyponatremia (Figure 2). Approximately, 10 days after TSS, the patient complained of blurred near vision.

In the ophthalmological examination before TSS, her vision was almost completely intact without visual field loss. The corrected visual acuity of both eyes was 20/20, the spherical equivalent of the right eye was −2.125 diopters, and that of the left eye was −2.0 diopters before TSS. However, 11 days after TSS, the spherical equivalent of the right eye had changed to −0.75 diopters, and that of the left eye had changed to −1.125 diopters. Figure 2 shows the time course of serum electrolyte and glucose levels, corneal radius, spherical equivalent, axial length, anterior chamber depth, and lens thickness of both eyes. Axial length, anterior chamber depth and lens thickness were calculated by A-mode ultrasonography. Lens power was calculated with Bennett’s formula [11] at the onset of blurred vision (11 days after surgery) and follow up (264 days), but could not be calculated before onset (i.e. before surgery and upto 10 days after) because we did not calculate axial length, anterior chamber depth and lens thickness over this time frame. There were no changes in axial length during follow up; however, both spherical equivalent and lens thickness were decreased, while in contrast the anterior chamber depth was increased. In addition, the size of both the decrease in lens thickness and the increase in anterior chamber depth was the same. The lens power (right eye: 17.52 diopters, left eye: 18.30 diopters) at onset increased to 20.27 diopters (right eye) and 20.11 diopters (left eye) at follow up (Table 1). Although her hyponatremia rapidly improved over a period of one week, the refractive change persisted for 6 weeks (43 days) after TSS and 4 weeks after onset. During her 7 months of follow-up, the level of serum glucose was not changed from a normal and the patient had no apparent recurrence. And also, the corrected visual acuity was not changed during the follow-up.

**Table 1:** Changing of the factors related to the refractive change

**At onset**	***d***_**1**_**: anterior chamber depth**	***d***_**1**_**: Lens thickness**	**Lens posterior Pole**	***F***_**L**_**: lens power**
RE	3.05 mm	4.21 mm	7.26 mm	17.52 diopters
LE	3.1 mm	4.03 mm	7.13 mm	18.30 diopters
At follow up				
RE	3.34 mm	3.89 mm	7.23 mm	20.27 diopters
LE	3.23 mm	3.9 mm	7.13 mm	20.11 diopters
Difference (onset minus follow up)				
RE	−0.29 mm	0.32 mm	0.03 mm	−2.75 diopters
LE	−0.13 mm	0.13 mm	0 mm	−1.81 diopters

## Discussion

Many ophthalmological reports have documented transient refractive change in systemic illness. However, this report is the first to present the hyperopia after TSS with hyponatremia. In DM, the main mechanism underlying refractive change has been implicated in which decreasing osmotic pressure of the aqueous humor causes consequent hydration of the lens [6-10]. In our patient, the average spherical equivalent of both eyes in the first visit was −2.06 diopters, at the time of initial complaint of blurred near vision was −0.95 diopters, and at approximately 6 months after onset had returned to the initial level. During the same time period, the average lens thickness of both eyes upon onset was 4.1 mm, and decreased to 3.9 mm 6 months after onset, implying that the lens was swelling and reverted to the initial thickness by 6 months. The patient exhibited no change in axial length throughout the studied time course. The most important fact in this case was that hyperopia and hyponatremia occurred simultaneously after TSS.

We found increased lens thickness and hyperopic shift after the hyponatremia in the present study. There are some concerns about the relationship between the refractive change and the related factors. Generally, increased lens thickness alone should result in a myopic shift.

Firstly, we need to consider the ciliary processes and the position of the lens. The anterior shift of the lens-iris diaphragm was able to cause myopia [12]. The posterior pole of the lens calculated as anterior chamber depth + lens thickness did not change during the study period (Table 1). The increase in lens thickness caused by hyponatremia matches the decrease in anterior chamber depth. If the lens swelling in its position is sustained by the zonule and the ciliary processes, the amount of a backward movement of the posterior pole could be at least equal to half the increase in lens thickness. With increasing age, the posterior pole recedes a little and the anterior chamber decreases by a similar amount leaving the lens in its usual position [13]. However, the present case showed that the posterior pole of the lens did not recede, suggesting that the refractive change was not due to changes in lens position.

Next, we consider both the corneal power and lens power during follow up. The corneal power was not changed. In contrast, the lens power at onset was less than during follow up according to Bennett’s formula (Table 1), suggesting that the lens has lost the power with swelling.

## Conclusions

Preoperative ophthalmologic examination before TSS is common. Routine examinations measure visual acuity, visual fields, and ocular pressure. However, we rarely get the opportunity to evaluate lens thickness and axial length. In this case, the hyperopic shift was caused by an increase in the lens thickness. Factors such as lens thickness and axial length should be taken into account when conducing preoperative ophthalmologic examinations especially for patients undergoing TSS.

### Consent

Written informed consent was obtained from the patient for publication of this Case Report and any accompanying images. A copy of the written consent is available for review by the Editor of this journal.

## Abbreviations

TSS: Trans-sphenoidal surgery; DM: Diabetes mellitus; MRI: Magnetic resonance imaging.

## Competing interests

The authors declare that we have no competing interests.

## Author’s contributions

HI conceived of the study and drafted the manuscript. JA critically revised the manuscript. KU performed the neurosurgery, collected the data, and helped to draft the manuscript. NI, TI, and CVB participated in the design of the study and helped to draft the manuscript. OM was involved in drafting the manuscript and participated in its design and coordination, and has given final approval of the version to be published. All authors read and approved the final manuscript.

## Pre-publication history

The pre-publication history for this paper can be accessed here:

http://www.biomedcentral.com/1471-2415/13/65/prepub
